# Adverse ventricular–ventricular interactions in right ventricular pressure load: Insights from pediatric pulmonary hypertension versus pulmonary stenosis

**DOI:** 10.14814/phy2.12833

**Published:** 2016-06-14

**Authors:** Mieke M. P. Driessen, Wei Hui, Bart H. Bijnens, Andreea Dragulescu, Luc Mertens, Folkert J. Meijboom, Mark K. Friedberg

**Affiliations:** ^1^ Department of Pediatric CardiologyThe Labatt Family Heart CentreThe Hospital for Sick ChildrenTorontoCanada; ^2^ Interuniversity Cardiology Institute of the Netherlands‐Netherlands Heart Institute (ICIN‐NHI)Utrechtthe Netherlands; ^3^ ICREAUniversitat Pompeu Fabra, Barcelona, Spain and KU LeuvenLeuvenBelgium; ^4^ Department of CardiologyUniversity Medical Center UtrechtUtrechtthe Netherlands

**Keywords:** Pediatrics, pulmonary hypertension, pulmonary stenosis, ventricular–ventricular interaction

## Abstract

Right ventricular (RV) pressure overload has a vastly different clinical course in children with idiopathic pulmonary arterial hypertension (iPAH) than in children with pulmonary stenosis (PS). While RV function is well recognized as a key prognostic factor in iPAH, adverse ventricular–ventricular interactions and LV dysfunction are less well characterized and the pathophysiology is incompletely understood. We compared ventricular–ventricular interactions as hypothesized drivers of biventricular dysfunction in pediatric iPAH versus PS. Eighteen iPAH, 16 PS patients and 18 age‐ and size‐matched controls were retrospectively studied. Cardiac cycle events were measured by M‐mode and Doppler echocardiography. Measurements were compared between groups using ANOVA with post hoc Dunnet's or ANCOVA including RV systolic pressure (RVSP; iPAH 96.8 ± 25.4 mmHg vs. PS 75.4 ± 18.9 mmHg; *P* = 0.011) as a covariate. RV‐free wall thickening was prolonged in iPAH versus PS, extending beyond pulmonary valve closure (638 ± 76 msec vs. 562 ± 76 msec vs. 473 ± 59 msec controls). LV and RV isovolumetric relaxation were prolonged in iPAH (*P* < 0.001; LV 102.8 ± 24.1 msec vs. 63.1 ± 13.7 msec; RV 95 [61–165] vs. 28 [0–43]), associated with adverse septal kinetics; characterized by rightward displacement in early systole and leftward displacement in late RV systole (i.e., early LV diastole). Early LV diastolic filling was decreased in iPAH (73 ± 15.9 vs. PS 87.4 ± 14.4 vs. controls 95.8 ± 12.5 cm/sec; *P* = 0.004). Prolonged RVFW thickening, prolonged RVFW isovolumetric times, and profound septal dyskinesia are associated with interventricular mechanical discoordination and decreased early LV filling in pediatric iPAH much more than PS. These adverse mechanics affect systolic and diastolic biventricular efficiency in iPAH and may form the basis for worse clinical outcomes. We used clinically derived data to study the pathophysiology of ventricular–ventricular interactions in right ventricular pressure overload, demonstrating distinct differences between pediatric pulmonary arterial hypertension (iPAH) and pulmonary stenosis (PS). Altered timing of right ventricular free wall contraction and profound septal dyskinesia are associated with interventricular mechanical discoordination and decreased early LV filling in iPAH much more than PS. These adverse mechanics affect systolic and diastolic biventricular efficiency, independent of right ventricular systolic pressure.

## Introduction

In both idiopathic pulmonary arterial hypertension (iPAH) and pulmonary valvular stenosis (PS) the right ventricle (RV) increases its systolic pressure in order to overcome the increased impedance. However, pediatric iPAH is associated with worse exercise capacity, morbidity, and mortality compared to PS (Haddad et al. 2008; Haworth and Hislop 2009). While RV morphology and dysfunction are well‐established drivers of morbidity and mortality in iPAH, and are different from PS (van Wolferen et al. 2007; Peacock et al. 2014), adverse ventricular–ventricular interactions and left ventricular (LV) dysfunction are emerging as important determinants of iPAH outcomes (Hardegree et al. 2013).

The RV and left ventricle (LV) are interdependent through common myocardial fibers, the interventricular septum (IVS) and the pericardial space (Belenkie et al. 1995; Friedberg and Redington 2014). Of these, the IVS plays a crucial role in mediating ventricular–ventricular interactions as it shares fibers with both ventricles, is subject to interventricular pressure gradients and directly impacts biventricular geometry (Santamore et al. 1976b; Buckberg 2006). Septal and LV contraction contribute substantially to RV systolic function and ultimately cardiac output (Santamore et al. 1976a; Klima et al. 1998). Likewise, RV loading, geometry, and function impact LV function (Santamore et al. 1976b; Dohi et al. 2008; Marcus et al. 2008; Hardegree et al. 2013). In RV pressure overload, these adverse ventricular–ventricular interactions negatively impact LV filling, geometry and systolic function (Dohi et al. 2008; Marcus et al. 2008; Hardegree et al. 2013). The mechanisms and pathophysiology of these adverse interactions is incompletely characterized, but may partly explain the different clinical course of these groups (Friedberg and Redington 2014). Comparing ventricular–ventricular interactions in pediatric iPAH versus PS can further our understanding of RV adaptation to increased pressure load.

Accordingly, the aim of this study was to characterize the pathophysiology and mechanisms of ventricular–ventricular interactions and their impact on biventricular function in the pressure‐loaded RV in iPAH versus PS, using detailed analysis of septal kinetics and cardiac cycle‐timing events. We hypothesized that adverse septal kinetics and temporal mechanics are associated with worse ventricular–ventricular interactions in children with iPAH versus PS.

## Methods

Patients with echocardiography performed between 2004 and 2013 were retrospectively studied. iPAH patients were diagnosed according to the Dana Point guidelines – that is, a mean pulmonary arterial pressure of ≥25 mmHg at rest with a pulmonary vascular resistance of ≥3 Wood units and a pulmonary capillary wedge pressure of ≤15 mmHg (Barst et al. 2011). Patients with valvular PS were included if they had no associated intracardiac abnormalities with the exception of a small, hemodynamically insignificant, atrial, or ventricular septal defect. Healthy controls matched for age and BSA, were selected from a database consisting of volunteers or children evaluated for an innocent cardiac murmur with normal medical history, physical examination, and echocardiography. The Institutional Research Ethics Board approved the study.

### Echocardiography

Analysis was performed on digitally stored echocardiograms (SyngoDynamics, Siemens, Erlangen, Germany) by *a single* observer. In iPAH patients the first full functional echocardiogram at our institution was analyzed. Measurements were performed as suggested in the 2010 guidelines for the pediatric echocardiogram by Lopez et al. (Lopez et al. 2010). A full functional study includes the following 2‐D echocardiography views: parasternal short axis at midpapillary level (M‐mode & 2‐D echocardiography), standard apical four‐chamber, separate RV focused four‐chamber view, and apical two‐chamber. Pulsed and continuous wave Doppler interrogation was performed for all valves and Tissue Doppler Imaging (TDI) of RV free wall (RVFW), LV lateral wall, and interventricular septum (IVS).

### Echocardiographic dimensions

M‐mode RV and LV end‐diastolic (EDD) and end‐systolic dimensions (ESD) and LV ejection fraction (Teichholz et al. 1976) were measured from parasternal short‐axis views; and *Z*‐scores calculated using institutional values. The LV eccentricity index (lateral divided by anterior‐posterior LV diameter) was measured using a mid‐LV short‐axis view at end‐systole, early diastole, and end‐diastole (Ryan et al. 1985). RV end‐diastolic and end‐systolic areas were measured in the apical four‐chamber view to calculate the RV fractional area change (Lopez et al. 2010). Tricuspid annular planar systolic excursion (TAPSE) and TDI systolic velocity (s’) were measured as measurements of RV systolic performance.

### Biventricular and septal kinetics

**Figure 1:**
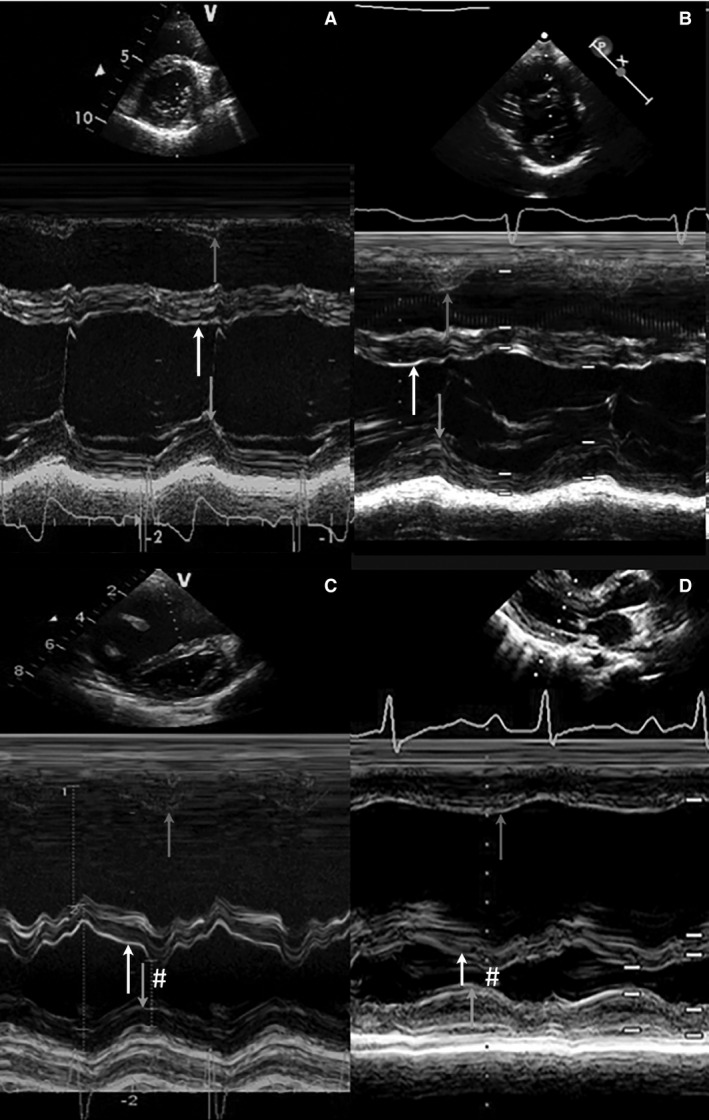
M‐mode cross sections. Four representative examples of m‐mode cross‐sectional parasternal short or long axis at the level of MV annulus are shown. The time to peak radial motion of right ventricular free wall, septum, and left ventricular posterior wall are depicted with arrows. (A) control, (B) pulmonary stenosis and (C and D) idiopathic pulmonary arterial hypertension. Abnormal septal motion in iPAH patients at the beginning of LV diastole is marked with # (C and D).

Detailed qualitative and quantitative analysis of IVS, RVFW and LV posterior wall (LVPW) motion throughout the cardiac cycle used parasternal midventricular M‐mode tracings to define septal motion in relation to the RVFW and LVPW. Pulsed wave Doppler echocardiography was used to measure valve events (i.e., valve opening and closure – detailed below) in relation to the mechanical events. Quantitative measurements included: time to onset and peak septal, RVFW and LVPW contraction and time to peak leftward septal displacement (when present; # Fig. 1). The electrocardiogram (ECG) QRS onset was used as a reference for timing and event measurements.

### Doppler echocardiography

Right ventricular systolic pressure (RVSP) was estimated from the TR jet using the modified Bernoulli equation. In PS patients the PV systolic gradient was used to estimate RVSP if the TR spectral Doppler tracing was incomplete. As inferior vena cava collapsibility is not validated or measured routinely in children, right atrial pressure was not added to the RVSP.

Pulsed Doppler tracings were sampled in the RV and LV inflow and outflow tracts. Atrio‐ventricular and ventriculo‐arterial valve opening and closure times and mitral and tricuspid valve early (E) and late (A) filling velocities were measured. LV isovolumetric relaxation time was derived from a pulsed wave Doppler tracing straddling the LV inflow and outflow.

Pulsed wave Tissue Doppler imaging (TDI) at the basal RV‐free wall, IVS and LV‐free wall was acquired and the peak systolic (TDI s’) and early diastolic (TDI e’) tissue velocities measured. The isovolumetric contraction and relaxation times were also measured on TDI.

An average of three measurements was used for all TDI and Doppler velocity measurements (Lopez et al. 2010). Timing measurements were taken from cardiac cycles with <10% variation in RR interval. To allow comparison between different heart rates, *all* timing measurements were normalized for the RR interval.

### Reproducibility

In 15 randomly selected patients all M‐mode and blood flow Doppler measurements including IVS, RVFW, and LVPW timing and valve events were remeasured by the first observer (with a 1 month interval) and by a second observer, blinded to the first measurement.

### Statistical analysis

Continuous variables are represented as mean ± standard deviation or median [range] as appropriate. Categorical variables are presented as a frequency (%). PS and iPAH were compared to controls using the ANOVA with post hoc Dunnet's or Mann–Whitney *U* test, as appropriate. To assess the effect of patient group on the dependent variable (outcome measurements) we used ANCOVA – as it enables analysis of a covariate that possibly confounds the analysis (in our case RVSP). In other words, if the relationship between patient group and the outcome variable is completely determined by RVSP – statistical analysis would yield a nonsignificant result for between group analysis.

Intra‐ and interobserver reproducibility was assessed by determining the mean difference with limits of agreement, intraclass correlation coefficient (absolute agreement) and comparing measurements with a paired Student's *t*‐test. An alpha level of 0.05 was considered statistically significant.

## Results

A total of 52 subjects, including 18 iPAH, 16 PS patients, and 18 age‐ and BSA‐matched controls were studied. Baseline and demographic data are listed in Table 1. Age, BSA, and sex were similar between the three groups. All iPAH patients were on pulmonary vasodilator treatments at time of echocardiography: 7 (39%) on phosphodiesterase inhibitors, 13 (72%) on endothelin‐receptor antagonists and 9 (50%) on prostacyclins. iPAH patients had significantly higher resting heart rates compared with controls. ECG QRS duration was significantly longer in iPAH versus PS patients and controls, although none met criteria for complete RBBB. RVSP and RV:LV pressure ratio were higher in iPAH patients compared with PS. iPAH patients had significantly more RV dilatation compared with controls and with PS patients; and measures of regional longitudinal and global RV function were worse (all *P* < 0.001; Table 1).

**Table 1:** Baseline characteristics

	Controls (*n* = 18)	PS (*n* = 16)	iPAH (*n* = 18)	*P*‐value PS versus iPAH
Age (Yrs.)*	11.2 ± 5.0	10.3 ± 4.7	11.5 ± 5.6	0.736
BSA (m^2^)*	1.31 ± 0.44	1.23 ± 0.48	1.16 ± 0.41	0.547
Male sex (%)	10 (56%)	9 (56%)	9 (50%)	0.716
Heart rate (bpm)	70 ± 13	81 ± 18	89 ± 22#	0.261
QRS ECG (msec)	–	84 ± 11	99 ± 18	0.005
RVSP (mmHg)	–	75.4 ± 18.9	96.8 ± 25.4	0.011
RV:LV pressure	–	0.71 [0.41–1.57]	1.10 [0.46–1.50]	0.007
RVDd (mm) *Z*‐score	18.5 ± 4.4 0.24 ± 1.02	20.3 ± 5.2 1.2 ± 1.56	34.4 ± 9.9* 4.82 ± 1.99*	<0.001 <0.001
RVDbas (mm)	33.5 ± 5.0	34.4 ± 7.4	48.3 ± 10.0*	<0.001
RVDmaj (mm)	60.4 ± 10.9	56.4 ± 11.2	66.6 ± 12.3	0.010
FAC (%)	47.2 [39.2–53.7]	42.5 [36.7–68.1]	18.5 [8.1–34.3]*	<0.001

Patients were compared to controls using ANOVA with post hoc Dunnet's test ^#^ *P* = 0.002 and *P < 0.001. PS and iPAH were compared using independent Student *T*‐test.

PS, pulmonary stenosis; iPAH, idiopathic pulmonary arterial hypertension; Yrs., years; BSA, body surface area; bpm, beats per minute; RVSP, right ventricular systolic pressure; LV, left ventricular; RVDd, right ventricular diastolic dimension parasternal long‐axis; RVDbas, basal AP4CH‐dimension; RVmajor, long AP4CH‐dimension; FAC, fractional area change.

### LV dimensions and functional parameters

Measures of LV dimensions and function are listed in Table 2. In PS patients these were comparable to controls. Using linear regression to account for differences in RVSP, iPAH patients had smaller LV EDD and LV ESD compared with PS patients and controls. LVEF was higher in iPAH. Both PS and iPAH patients had lower MV E/A ratio and similar E/E’ ratio than controls. The early diastolic LV filling velocity was lower in iPAH compared with controls and PS patients (*P* < 0.001 and *P* = 0.004, respectively).

**Table 2:** LV dimensions and global function

	Controls (*n* = 18)	PS (*n* = 16)	iPAH (*n* = 18)	*P*‐value iPAH to PS
LVEDd (mm) *Z*‐score	42.6 ± 6.6 −0.01 ± 1.05	39.2 ± 7.3 −0.53 ± 1.12	31.4 ± 8.1* −3.86 ± 3.27*	0.101 0.011
LVEDs (mm) *Z*‐score	26.2 ± 4.6 −0.24 ± 0.85	23.6 ± 4.9 −0.82 ± 1.14	16.9 ± 6.9* −3.70 ± 3.27*	0.052 0.029
LVEF‐teich (%)	76.1 ± 6.0	77.2 ± 7.3	83.7 ± 8.7**	0.032
E vel (cm/sec)^a^	95.8 ± 12.5	87.4 ± 14.4	73.9 ± 15.9*	0.004
MV E/A ratio^a^	2.66 ± 0.90	1.70 ± 0.56*	1.44 ± 0.40*	0.594
MV E/E’ ratio^b^	5.0 [4.3–9.1]	5.1 [4.1–7.1]	6.1 [4.3–9.9]	0.238
Sys ecc index	1.07 ± 0.03	1.23 ± 0.15#	2.49 ± 0.96*	<0.001
BDia ecc index^c^	1.17 ± 0.05	1.38 ± 0.16**	3.23 ± 1.22*	<0.001

Controls compared with pulmonary stenosis (PS) and idiopathic pulmonary arterial hypertension (iPAH) patients using ANOVA with post hoc Dunnet's; *P ≤ 0.001, **P ≤ 0.01 and ^#^ *P* < 0.05. Patient groups are compared using ANCOVA including right ventricular systolic pressure as a covariate.

LV, left ventricular; EDd, end‐diastolic dimension; EF, ejection fraction; E, early diastolic filling velocity; A, late diastolic filling velocity; ecc, eccentricity index; Sys, systolic; Bdia, begin diastolic.

a total fusion in 1 iPAH, missing in 4 iPAH and 1 PS patient.

b missing in 6 iPAH and 2 PS patients.

c Eccentricity index in early diastole.

### Event timing and septal movement

**Figure 2:**
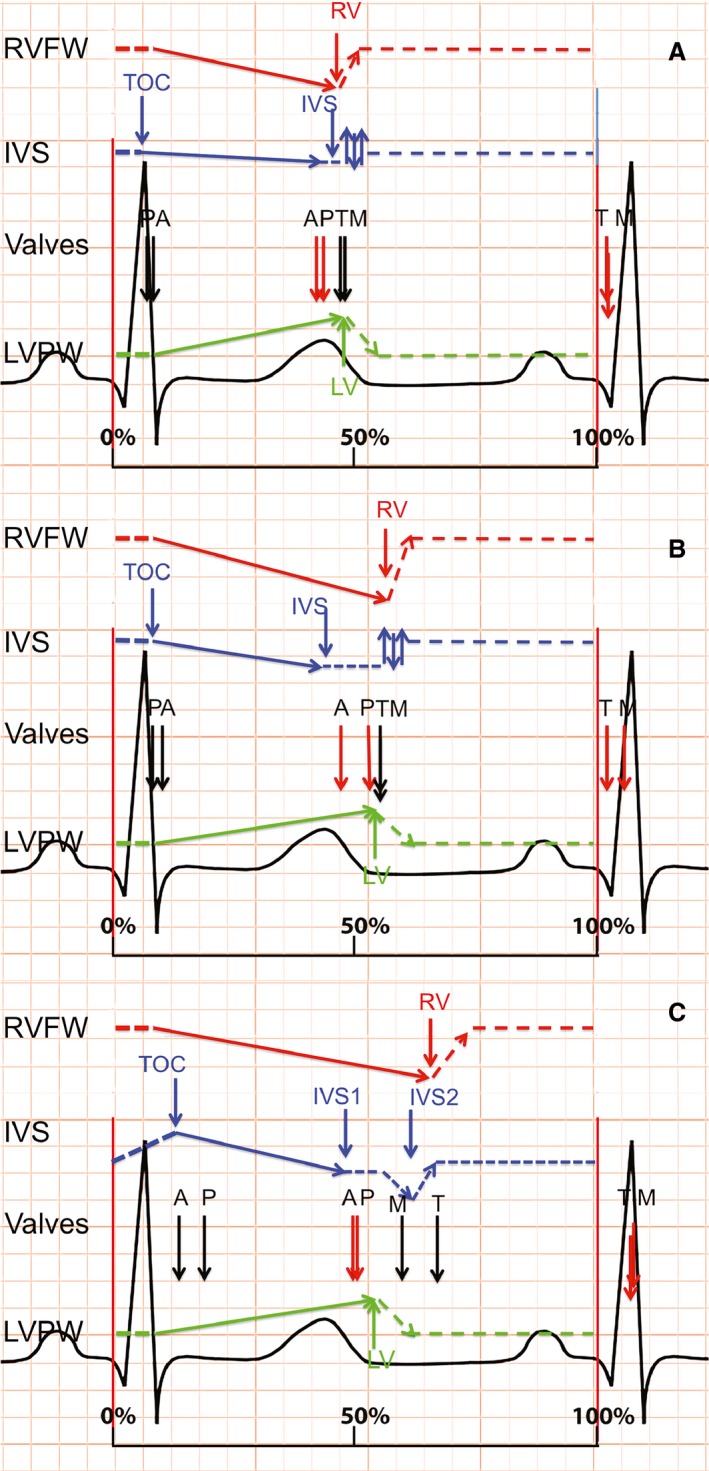
Schematic representation of cardiac events. Schematic representation of peak contraction and valve timing (opening in black and closure in red), relative to RR interval, for each group. A = controls; B = pulmonary stenosis; C = idiopathic pulmonary arterial hypertension. TOC, time of onset contraction; IVS, interventricular septum; LV, left ventricular free wall; RV, right ventricular free wall; A, aortic valve; P, pulmonic valve; M, mitral valve; T, tricuspid valve.

Figure 1 shows representative M‐mode examples of each group. In Figure 2 the average timing of peak contraction and valve timing are represented schematically, including only patients with a complete dataset.

#### Healthy controls

Normal IVS motion is characterized by three major components (Figs. 1A and 2A): (1) the IVS starts to contract on average 58 ± 3 msec after QRS‐onset, maintaining a stable position throughout systole; (2) Peak IVS systolic excursion occurs ~30 msec before peak RVFW and LVPW thickening, which peak near‐simultaneously (Table 3); (3) During early RVFW and LVPW relaxation there is subtle bidirectional postsystolic IVS motion (# in Fig. 1A). The pulmonary valve (PV) opens slightly before, and closes simultaneously with, the aortic valve (AV). The mitral (MV) and tricuspid valves (TV) open and close near‐simultaneously (Figs. 1A and 2A).

**Table 3:** Timing of contraction and valve opening and closure

	Controls (*n* = 18)	PS (*n* = 16)	iPAH (*n* = 18)	*P*‐value iPAH to PS
RVFW peak (msec)^a^	473 ± 59	562 ± 76**	638 ± 76*	0.031
LVPW peak (msec)^a^	478 ± 48	538 ± 70#	564 ± 80*	0.547
Septal peak (msec)^a^	442 ± 74	435 ± 70	478 ± 51	0.212
Septal‐D peak*, a	–	–	613 ± 63	–
TOC (msec)^a^	58 ± 31	91 ± 24**	122 ± 36*	0.003
PV opening (msec)^b^	66 ± 12	81 ± 29	178 ± 76*	0.001
PV closure (msec)^b^	445 ± 52	514 ± 68*	500 ± 73	0.079
ET RVOT (msec)	374 ± 48	451 ± 60*	364 ± 62	0.001
AV opening (msec)^b^	83 ± 14	95 ± 15#	130 ± 40*	0.15
AV closure (msec)^b^	433 ± 52	461 ± 57	490 ± 63#	0.923
ET LVOT (msec)	354 ± 44	362 ± 46	375 ± 45	0.538
MV opening (msec)^c^	492 ± 52	537 ± 69#	592 ± 90*	0.520
TV opening (msec)^c^	478 ± 55	541 ± 88#	667 ± 98*	0.04
IVRT LV (msec)	63 ± 14	73 ± 16	103 ± 24*	0.015
TDI IVCT RV (msec)	70 [61–102]	61 [48–85]	90 [58–139]**	<0.001
TDI IVRT RV (ms)	28 [0–43]	33 [24–123]	95 [61–165] *	<0.001
MV inflow (msec)^c^	545 ± 49	531 ± 63	482 ± 82#	0.827
TV inflow (msec)^c^	557 ± 62	484 ± 87	399 ± 88*	0.251

Timing measurements normalized for the RR interval, patients compared with controls using ANOVA with Dunnet's; ^#^ *P* < 0.05, *P < 0.01, **P < 0.001. Pulmonary stenosis (PS) are compared to idiopathic pulmonary arterial hypertension (iPAH) patients using ANCOVA using right ventricular systolic pressure as a covariate.

RVFW, Right ventricular free wall; LVPW, Left ventricular posterior wall; D, diastolic peak; TOC, time to onset of contraction; PV, pulmonary valve; AV, aortic valve; MV, mitral valve; TV, tricuspid valve; IVRT, isovolumetric relaxation time.

a RVFW peak missing in 4 controls, 2 PS and 1 iPAH patients, these were excluded for all M‐mode measurements.

b VA valve opening missing in 6 iPAH and 1 PS patient.

c AV opening was missing in 5 iPAH and 1 PS patient.

#### PS

In PS, qualitative IVS motion is similar to controls with some differences in the timing and position of the IVS: Onset of IVS contraction is delayed compared to controls (90 ± 23 msec vs. 58 ± 3 msec; *P* < 0.01), with a corresponding mild delay in AoV opening (95 ± 15 msec vs. 83 ± 14 msec; *P* < 0.05) which coincides with PV opening. Peak IVS systolic excursion and the early diastolic bidirectional IVS motion (Fig. 1A and B) are similar to controls. Peak RVFW and LVPW thickening are delayed compared with controls but remain near simultaneous (peak RVFW 562 ± 76 and LVPW 538 ± 70 msec Table 3, Figs. 1B and 2B). This results in prolonged mechanical discoordination between the IVS and both free walls (IVS to LVPW delay 121 msec) compared to controls. The septum is mildly leftward displaced during systole. Peak RVFW thickening is slightly later than peak LVPW thickening; correspondingly the PV closes later than the AoV with a prolonged RV ejection time (514 ± 68 msec vs. 461 ± 57 msec in controls). As systole lasts longer in PS compared to controls, MV and TV opening are delayed in PS despite normal isovolumetric relaxation times. The opening of the MV and TV valves is simultaneous.

#### iPAH

Qualitatively and quantitatively septal motion and timing of biventricular contraction and relaxation in iPAH patients differ substantially from PS patients and controls (Fig. 1A–D). Onset of IVS contraction is delayed to 122 ± 36 msec after QRS‐onset (PS 90 ± 23; controls 58 ± 3 msec; *P* < 0.01). The IVS is displaced toward the RVFW during this delay and RVFW isovolumetric contraction is prolonged (iPAH 90 [58–139] vs. PS 61 [48–85] & controls 70 [61–102] msec; *P* < 0.01). The delayed onset of effective IVS and RVFW contraction corresponds with a delay in both PV and AoV opening compared to PS and controls (Table 3). In contrast to controls and PS, the PV opens after the AoV. In contrast to PS and controls the IVS thickens only mildly and is displaced markedly toward LVPW throughout systole (Figs. 1 and 2), reflected by the high LV eccentricity index (Table 2). Similar to PS, both RVFW and LVPW peak thickening are prolonged compared to controls. Peak RVFW thickening is more prolonged in iPAH versus PS (iPAH 638 ± 76 vs. PS 562 ± 76 and controls 473 ± 59 msec; *P* < 0.05), extending well beyond peak LVPW thickening and PV closure (564 ± 80 & 500 ± 73 msec, respectively) resulting in LV‐RV systolic discoordination and postsystolic RVFW thickening. Despite prolonged RVFW thickening, effective RV ejection time is short (364 ± 62 msec vs. 374 ± 48 msec in controls). During postsystolic RVFW thickening, the IVS displaces even further leftward (# Figs. 1C, D and 2C), persisting throughout diastole. LV and RV isovolumetric relaxation times are prolonged in iPAH compared with PS and controls. Both MV and TV opening are delayed and discoordinated compared to controls and PS – the TV opening much later than the MV. The MV opens whilst the septum is still displaced toward the LVPW (MV opening 592 ± 90 vs. peak IVS displacement 613 ± 63 msec). This is associated with decreased early mitral diastolic filling velocity (E) compared to PS and controls (73.9 ± 15.9 vs. 87.4 ± 14.4 and 95.8 ± 12.5 cm/sec; *P* = 0.004) and both LV and RV inflow durations are significantly shortened compared with controls (but not PS).

#### Subgroup analysis similar RVSP

A subanalysis was performed in patients with comparable RVSP (50–110 mmHg); 11 iPAH patients and 15 PS patients could be included, with mean RVSP of 83.8 ± 18.6 mmHg versus 77.4 ± 17.6 mmHg (*P* = 0.387). Differences in timing between iPAH and PS patients were similar to those observed in the entire cohort; time to peak RVFW thickening was delayed in iPAH (630 ± 82 msec vs. 562 ± 76 msec; *P* = 0.042), ending well after PVC (477 ± 82 msec vs. 514 ± 70 msec in PS) and peak LVPW thickening (566 ± 81 msec vs. 538 ± 69 msec; *P* = 0.369). The time to onset of IVS contraction was prolonged in iPAH versus PS patients (123 ± 38 msec vs. 90 ± 24 msec; *P* = 0.015), moving rightward during this interval. All iPAH patients showed IVS displacement (#) after peak LVPW thickening (611 ± 67 vs. peak LVPW 566 ± 81 msec). In PS patients IVS displacement was only seen during end‐systole, preceding or coinciding with peak LVPW thickening.

## Discussion

Our results show that adverse ventricular–ventricular interactions and septal displacement are markedly worse in RV pressure load associated with pediatric iPAH compared to PS. Pediatric iPAH was associated with profound RV early and end systolic (i.e., early LV diastolic) septal displacement and biventricular mechanical discoordination in both systole and diastole – reducing contractile efficiency and early LV filling. In contrast, in PS LV‐RV coordination was preserved in both in systole and diastole and only mild septal displacement was seen at end‐systole. These adverse v‐v interactions seem primarily related to altered timing of RVFW systolic events seen in iPAH more than PS, and attest to both RV inefficiency and LV diastolic compromise.

### Systolic interaction

Septal position depends on the transseptal pressure gradient combined with the tendency of the septum to straighten when contracting (Kingma et al. 1983). Changes in RV load therefore influence septal configuration and end‐systolic septal curvature was one of the first sensitive, noninvasive markers of RV pressure overload (Santamore et al. 1976b; Kingma et al. 1983; Ryan et al. 1985). Furthermore, adult iPAH studies have related septal kinetics to disease severity (Mori et al. 2008; Sciancalepore et al. 2012). However, studies detailing IVS kinetics throughout the cardiac cycle remain very limited. Our results extend those of previous studies and demonstrate abnormal septal kinetics in pediatric iPAH patients throughout the entire cardiac cycle, whereas those in PS were limited to end‐systole. In iPAH pronounced septal displacement was not only seen in late RV systole (prolonged RVFW thickening) – toward the LVPW – but also during early systole – toward the RVFW (# Figs. 1C, D and 2C).

Abnormal iPAH septal motion coincided with differences in timing of RVFW systolic events in iPAH versus PS patients. First, iPAH patients have prolonged RV isovolumetric contraction (median 90 msec vs. 61 msec in PS) – and hence prolonged pressure generation – coinciding with the early systolic rightward septal displacement. This delays not only effective RV, but also LV ejection in iPAH. Secondly, prolonged peak RVFW systolic excursion is delayed in iPAH much more than PS (mean of 638 msec vs. 562 msec) and results in end‐systolic LVPW‐RVFW‐IVS mechanical discoordination and postsystolic thickening *only* in the iPAH group. Previous studies in adults described the negative impact of end‐systolic RV‐LV dyssynchrony on RV systolic function (Lopez‐Candales et al. 2005; Marcus et al. 2008). During end‐systolic discoordination – RV work is inefficiently spent on displacing the septum leftward rather than ejecting blood – thereby decreasing RV pump efficiency (Fig. 2). Moreover, despite the delayed peak RVFW thickening also observed in PS patients, their isovolumetric times remain short and RVFW and LVPW remain coordinated, without impediment of RV systolic function.

### Diastolic interactions

Early LV filling was decreased in our pediatric iPAH population, but was normal in PS when compared to controls (Table 2). Additionally, isovolumetric relaxation times were prolonged in iPAH resulting in pronounced delay of MV and TV opening and also diastolic discoordination. Previous studies attributed abnormal LV filling in adult iPAH, to both direct ventricular–ventricular interaction, that is, septal displacement, or in‐series interaction, that is, LV underfilling due to decreased RV cardiac output (Santamore et al. 1976b; Belenkie et al. 1995; Marcus et al. 2001, 2008; Gan et al. 2006; Lumens et al. 2010). Contrary to our findings, Lurz et al. demonstrated similar results in patients with RV pressure overload in the context of congenital heart disease (Lurz et al. 2009). We observed marked septal displacement – that is, direct interaction – in iPAH but not PS during early LV diastole (Fig. 2), occurring at time of mitral valve opens – impeding filling. Likewise, pediatric iPAH patients exhibited both postsystolic RVFW thickening and leftward septal displacement, both previously associated with RV systolic dysfunction and lower RV stroke volume (Lopez‐Candales et al. 2005; Marcus et al. 2008; Lumens et al. 2010). In addition, our pediatric iPAH cohort had markedly remodeled RVs. Santamore et al. showed that increased RV end‐diastolic volume independently alters LV diastolic pressure‐volume relations, decreasing LV filling (Santamore et al. 1976b). Lastly, in contrast to PS and controls – there was a marked delay between MV and TV opening (592 msec vs. 667 msec after QRS‐onset) – which is a novel finding. In light of literature and our current results, it would seem that both in‐series and direct interactions are present (Baker et al. 1998).

### Differences between iPAH and PS

Septal kinetics and ventricular–ventricular interactions differed substantially between PS and iPAH. Peak systolic RV pressures were higher in pediatric iPAH versus PS. Although this contributes to the differences between the patient populations, RVSP was included as a covariate in our analysis and the results were consistent across the entire range of RVSP – rendering this an insufficient explanation in and of itself (Bogaard et al. 2009). As outlined above – altered timing of RVFW systolic events seems to be the main driver of adverse ventricular–ventricular interactions and septal displacement. The prolonged RV isovolumetric contraction time might partly relate to the longer QRS duration (mean 99.4 msec vs. 83.9 msec in PS), but seems insufficient to explain the large dispersion in peak RVFW thickening (638 msec in iPAH vs. 562 msec in PS). Irrespective of peak RV systolic pressures, afterload as defined by end‐systolic wall stress, may be higher in iPAH versus PS – in analogy with LV data, thereby resulting in a higher RV load in iPAH (Borow et al. 1985). RV load in iPAH patients might be even further augmented by factors such decreased capacitance and reflected waves. The marked RV dilation in iPAH also contributes to septal displacement and decreased early LV filling – both directly and as a result of confinement within the common pericardial space (Santamore et al. 1976b; Belenkie et al. 2004). Evidence of different RV adaptation to congenital lesions – such as PS, versus – later onset – iPAH is growing and the subject of separate studies investigating myocardial remodeling (Bogaard et al. 2009). Furthermore, changes in RV afterload also alter IVS stress, possibly triggering adverse myocardial remodeling – with increased myocardial fibrosis (Sanz et al. 2007). In combination, with the adverse hemodynamics detailed above, this would be expected to worsen the biventricular inefficiency observed in this study.

### Clinical implications

Pulmonary vasodilators are the cornerstone of iPAH treatment and – by lowering pulmonary vascular resistance – decrease wall stress, improve transseptal gradient and thus ventricular–ventricular interactions. However, despite combination therapy, RV pressure often remains elevated as observed in our iPAH cohort. Our data suggest that improvement of the timing of contraction, to decrease mechanical LVPW and RVFW discoordination, may be a worthwhile therapeutic target in iPAH. This may be achieved electro‐mechanically – via pacing – or medically – by reversing myocardial remodeling that causes mechanical delays. Both approaches have been explored. Lumens et al. investigated RV pacing in a computer model of iPAH‐RV failure and demonstrated improved RV end‐diastolic volumes, RVFW myofiber work and pump function through improved distribution of workload (Lumens et al. 2009). Likewise, RV pacing improved LV diastolic filling in experimental isolated hearts and in an observational human study of chronic thrombo‐embolic PAH (Handoko et al. 2009; Hardziyenka et al. 2011). We previously showed that pharmacological intervention can also modulate adverse ventricular–ventricular interactions. In a rat model of iPAH, carvedilol improved biventricular remodeling, myocardial fibrosis and improved LV and RV Tau – shortening biventricular isovolumetric periods (Okumura et al. 2015).

### Study limitations

This was a retrospective study with inherent limitations. Doppler data were missing in a small number of patients. Likewise, invasive hemodynamic data were not consistently available at time of echocardiography. iPAH remains uncommon in children and the sample size is relatively small. However, the observations were consistent between patients. We were unable to provide clinical outcomes or exercise data. Although this is a drawback of the retrospective nature of our study – differences in natural history between iPAH and PS have been well described and we emphasized the mechanics and pathophysiology underlying these known differences.

## Conclusion

Prolonged RVFW thickening, prolonged isovolumetric times and profound early and late systolic septal displacement are related to RV mechanical inefficiency, interventricular mechanical discoordination, and decreased early LV filling in pediatric iPAH patients. These were absent in PS – suggesting that increased RVSP in itself is insufficient to explain the differences. Our findings show that iPAH affects systolic and diastolic mechanics in the right and left ventricles, and that the interaction between them is a prominent component of abnormal mechanics.

## Conflict of Interest

None declared.
